# Autosomal dominant inheritance of a heterozygous mutation in *HTRA1*: A case report and literature review

**DOI:** 10.1097/MD.0000000000048528

**Published:** 2026-05-08

**Authors:** Jianzhong Shu, Jinrong Li

**Affiliations:** a Encephalopathy Department, Chongqing Traditional Chinese Medicine Hospital, Chongqing, China.

**Keywords:** autosomal dominant disorder, case report, cerebral small-vessel disease, HTRA1 gene with heterozygous mutation

## Abstract

**Rationale:**

Heterozygous mutations in the gene encoding HtrA serine protease 1 (HTRA1) can cause autosomal dominant cerebral small-vessel disease (CSVD), presenting with clinical features that differ slightly from other hereditary CSVD subtypes. Only a limited number of cases have been documented globally.

**Patient concerns:**

A 51-year-old man presented with more than 2 years of left lower limb weakness, which had acutely worsened over the preceding day. Brain magnetic resonance imaging (MRI) revealed ischemic demyelinating changes in the white matter.

**Diagnoses:**

Genetic sequencing identified a heterozygous HTRA1 mutation. Follow-up testing confirmed that both of his daughters also carried the same heterozygous mutation.

**Interventions:**

Management focused on secondary prevention, risk-factor control, and lifestyle modification.

**Outcomes:**

A 6-month follow-up cranial MRI showed no new lesions, and the patient remained clinically stable without disease progression during 1 year of monitoring.

**Lessons:**

Heterozygous HTRA1 mutations may present as autosomal-dominant CSVD, and genetic testing is central to establishing the diagnosis.

## 1. Introduction

Hereditary forms of cerebral small-vessel disease (CSVD) have attracted considerable attention. Although homozygous mutations in the HtrA serine protease 1 (HTRA1) gene, which cause cerebral autosomal recessive arteriopathy with subcortical infarcts and leukoencephalopathy (CARASIL), are well recognized, reports of autosomal dominant CSVD resulting from heterozygous HTRA1 mutations remain relatively limited. This report presents a family with an autosomal dominant pattern of inheritance associated with a heterozygous HTRA1 mutation. A detailed description of the genetic findings and clinical features, along with a review of the relevant literature, is provided to support clinical diagnosis, genetic counseling, and therapeutic decision-making. By conducting an in-depth analysis of this family, we aim to contribute to the early recognition and management of this disorder and to offer a valuable case resource for researchers seeking to advance understanding and awareness of HTRA1-related disease.

## 2. Case presentation

**Figure 1.**
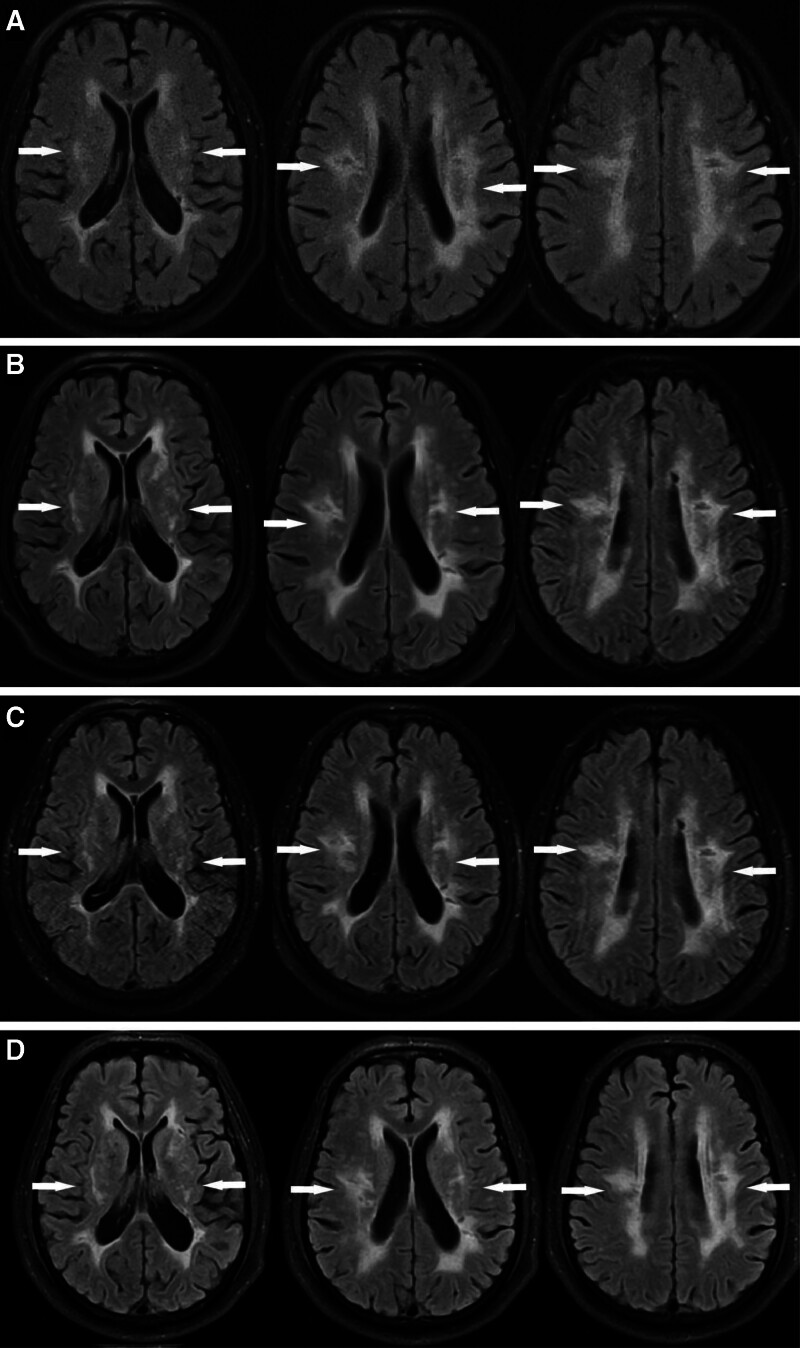
Brain MRI FLAIR order indicated high signals associated with the white matter near the bilateral ventricles. (A) 2021-10-02; (B) 2023-08-04; (C) 2024-02-21; (D) 2024-08-13. FLAIR = fluid-attenuated inversion recovery, MRI = magnetic resonance imaging.

A 51-year-old man was admitted to our hospital on February 20, 2024, with a chief complaint of left lower limb weakness persisting for more than 2 years and worsening over the preceding day. The patient initially developed unexplained weakness in the left lower limb relative to the right 2 years ago. His symptoms had recently progressed, accompanied by mild memory impairment. Two previous MRI scans performed at external institutions revealed high-signal changes in the periventricular white matter (Fig. 1A, B). He had not received treatment at that time, and his symptoms had remained stable without further deterioration. One day before admission, he experienced worsening weakness and claudication.

**Figure 2.**
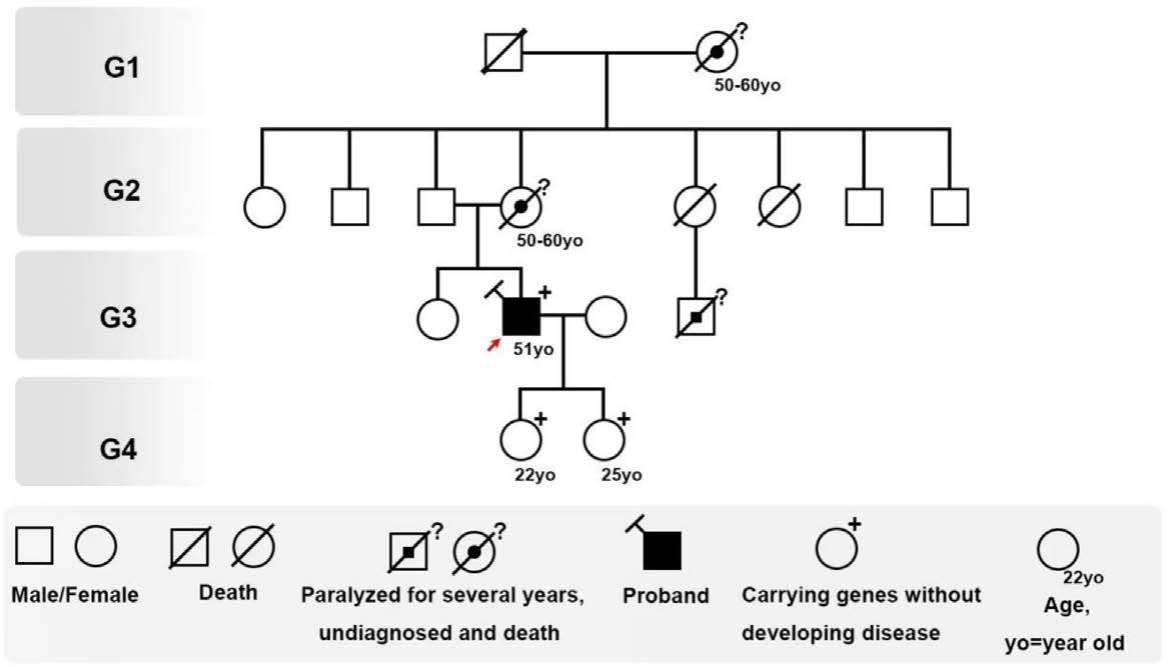
Pedigree.

The patient had no history of headaches and was otherwise in good health. He drank alcohol lightly and was married; he had 2 healthy adult daughters in their twenties. His family history was significant for the deaths of his mother, maternal grandmother, and a maternal cousin between the ages of 50 and 60; all had experienced progressive paralysis and prolonged bedridden states before death without a definitive diagnosis. The family pedigree is shown in Figure 2.

On physical examination, his vital signs were stable, and systemic findings were unremarkable, with no evidence of alopecia. Neurological evaluation revealed deficits in recent memory and computation. Muscle strength in the left lower limb was grade 5, and the left Babinski sign was positive, without additional focal findings. His NIH Stroke Scale score was 1 (left lower limb). Following admission, laboratory evaluation identified hyperlipidemia and type 2 diabetes. Cognitive assessment showed normal results on the Mini-Mental State Examination (MMSE) and Hasegawa’s Dementia Scale, while the Montreal Cognitive Assessment (MoCA) score was 15.

**Figure 3.**
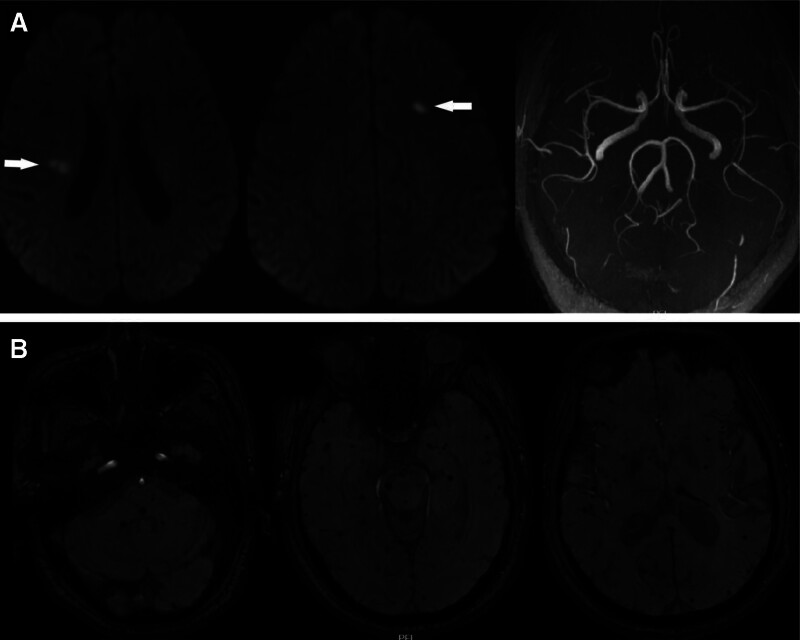
(A) DWI showed acute infarction foci in the right paraventricular body and left frontal lobe; MRA did not reveal any obvious abnormality. (B) SWI showed multiple microhemorrhagic foci/hemosiderin deposits in the brain parenchyma. DWI = diffusion-weighted imaging, MRA = magnetic resonance angiography, SWI = susceptibility-weighted imaging.

Brain MRI demonstrated scattered ischemic and infarcted lesions with ischemic demyelination of the white matter, increased in number compared with earlier scans (Fig. 1C). Diffusion-weighted imaging (DWI) showed acute infarcts in the right periventricular region and left frontal lobe. Magnetic resonance angiography was unremarkable. Susceptibility-weighted imaging (SWI) revealed multiple microhemorrhages and hemosiderin deposits throughout the brain parenchyma (Fig. 3).

**Figure 4.**
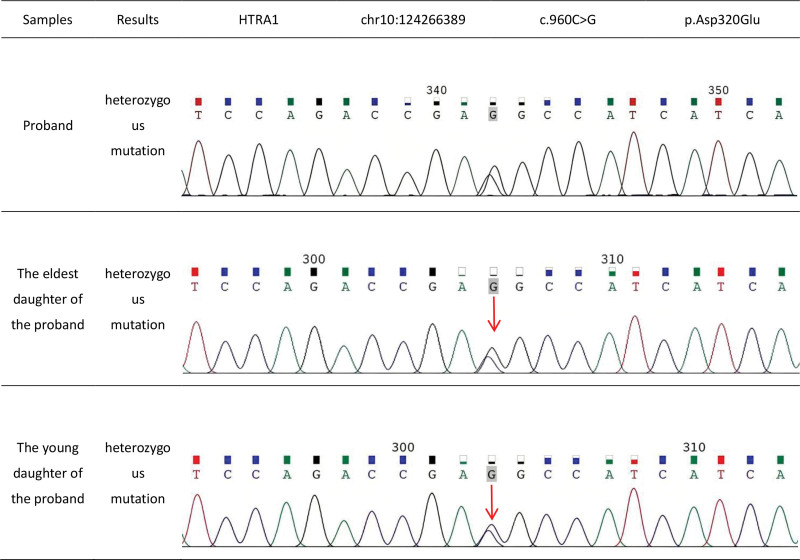
Genetic testing identified a heterozygous mutation in the gene encoding *HTRA1*. HTRA1 = HtrA serine protease 1.

Genetic testing using a white matter and cerebrovascular disease panel detected an *HTRA1* mutation at chr10:124266389, c.960C>G, p.Asp320Glu. This missense variant substitutes cytosine for guanine at nucleotide 960, replacing aspartate with glutamate at amino acid position 320 (Fig. 4). Both of the patient’s daughters carried the same heterozygous *HTRA1* mutation. A diagnosis of *HTRA1*-related CSVD was established.

**Figure 5.**
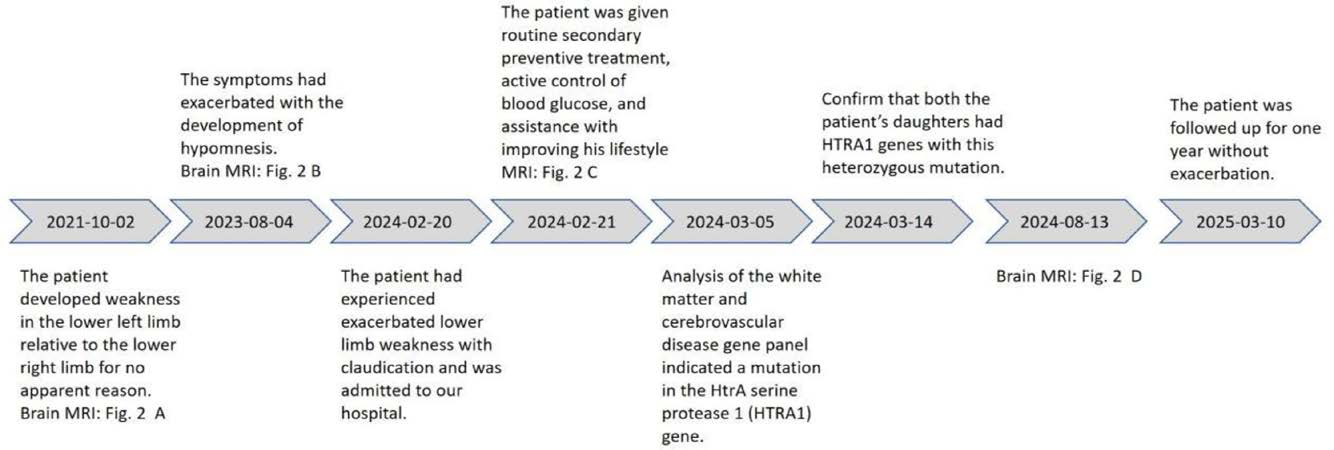
Timeline of disease development, diagnosis, and interventions. MRI = magnetic resonance imaging.

The patient received standard secondary preventive therapy, including aspirin (100 mg once daily), atorvastatin (20 mg once daily), and strict glycemic control. He was counseled on lifestyle modification, including cessation of harmful habits, maintenance of regular sleep patterns, and engagement in aerobic exercise 5 times/wk for 30 min/session after discharge. Once stabilized, he resumed work while avoiding excessive physical strain. Monthly outpatient follow-up included routine monitoring of liver and renal function, serum lipids, and glycated hemoglobin, all of which remained within normal ranges. Follow-up brain MRI and DWI showed no new lesions (Fig. 1D). Over 1 year of observation, the patient remained clinically stable without further exacerbations. A timeline summarizing the major disease milestones is provided in Figure 5.

## 3. Discussion

Hereditary cerebral small-vessel disease (CSVD) encompasses a group of rare cerebrovascular disorders caused by monogenic mutations.^[1]^ The first identified entity in this category was cerebral autosomal dominant arteriopathy with subcortical infarcts and leukoencephalopathy (CADASIL), which is attributed to mutations in the NOTCH3 gene on chromosome 19 and was first reported in 1996.^[2]^ In 2009, homozygous mutations in the *HTRA1* gene were recognized as the cause of cerebral autosomal recessive arteriopathy with subcortical infarcts and CARASIL.^[3]^ The *HTRA1* gene encodes HTRA1, a protein involved in extracellular matrix degradation, angiogenesis, and maintenance of vascular homeostasis. *HTRA1* also negatively regulates signaling by the transforming growth factor-β (TGF-β) family, which is essential for vascular smooth muscle cell differentiation. Excessive activation of TGF-β–related pathways can lead to degeneration of vascular smooth muscle cells. At the same time, upregulated TGF-β signaling has also been implicated in clinical manifestations such as alopecia and spinal abnormalities.^[4]^ Previous research on CARASIL indicates that individuals carrying heterozygous *HTRA1* mutations typically do not exhibit the classical features of the disorder, such as early-onset white matter disease, alopecia, or lumbago. This has led to the assumption that heterozygous carriers retain approximately 50% of normal *HTRA1* enzymatic activity, which appears sufficient to prevent the development of the full CARASIL phenotype.^[5]^

The patient described in this report developed symptoms in middle adulthood, demonstrating a stepwise progression of neurological deficits, without a history of headache or spinal disease, and with a family history consistent with autosomal dominant inheritance. Genetic testing identified a heterozygous *HTRA1* mutation, and both the clinical presentation and imaging findings differed substantially from those typically observed in CADASIL or CARASIL. Previous studies have shown that heterozygous *HTRA1* mutations can cause a rare CADASIL-like disorder with autosomal dominant transmission,^[6]^ for which the European Academy of Neurology has proposed the term *HTRA1*-autosomal dominant disorder (AD-HTRA1).^[7]^ Compared with CARASIL, AD-*HTRA1* is characterized by a milder phenotype, later onset, a higher prevalence of conventional vascular risk factors, more gradual clinical progression, and fewer extraneurological features, such as early-onset spinal disease and alopecia.^[8]^ The epidemiological profile, clinical manifestations, and imaging findings of this patient are consistent with these defining characteristics.

According to the available literature, AD-HTRA1 cases have been reported most frequently in China, with onset typically between 40 and 60 years of age. Clinical symptoms and imaging findings often resemble those of sporadic CSVD, contributing to the lack of disease specificity.^[9]^ To date, most reported cases of this disorder have originated from China.^[9]^ Outside China and East Asia, only a limited number of studies have been published. A French cohort study that screened CSVD-related genes in 3853 unrelated adults suspected of hereditary CSVD at Saint Louis Hospital in Paris identified 9 clinically significant heterozygous *HTRA1* mutations.^[10]^ Similarly, researchers in Turkey and India have reported heterozygous HTRA1 mutations in patients with ischemic stroke, with clinical features consistent with previously described phenotypes.^[11,12]^

Bioinformatics tools were used to assess the pathogenicity of the identified variant. SIFT predicted the mutation to “affect protein function,” while PolyPhen-2 classified it as “probably damaging.” Other predictive scores included BayesDel_noAF (0.34143), REVEL (0.806), and VEST4 (0.974). The missense-meta prediction result was “20/23.” According to ACMG guidelines, the variant met the criteria PM2_Supporting and PP3_Strong. Genetic testing of the patient’s 2 daughters confirmed the presence of the same heterozygous *HTRA1* mutation, supporting autosomal dominant inheritance. Both daughters are currently asymptomatic, and continued longitudinal follow-up will be required to assess the penetrance of this variant.

The c.960C>G (p.Asp320Glu) variant is absent from major population databases, including gnomAD, ExAC, and the 1000 Genomes Project, and is not listed in ClinVar. A PubMed search conducted on 2025-12-10 identified no publications referencing this variant, confirming its novelty (Table 1). This report broadens the known spectrum of heterozygous *HTRA1* mutations associated with autosomal dominant inheritance and provides additional clinical context for the characterization of CSVD in patients carrying heterozygous *HTRA1* variants.

**Table 1:** Summary of *HTRA1* c.960C>G (p.Asp320Glu) variant retrieval results in public databases.

Database name	Reference sequence/retrieval basis	Inclusion status	Key relevant information	Retrieval version/date
ClinVar (NCBI)	HTRA1: NM_002775.5; HGVS: c.960C>G	Not included	No corresponding variation ID; reported variant at the same protein position p.Asp320 is c.958G>A (p.Asp320Asn), which is distinct from this variant	2025-12
Genome Aggregation Database (gnomAD)	GRCh38 chr10:122489509; HTRA1 c.960C>G	Not included	No population allele frequency data; the variant was not detected in versions v2.1.1 and v3.1.2	v2.1.1/v3.1.2; 2025-12
dbSNP (NCBI)	HTRA1: NM_002775.5; HGVS: c.960C>G	Not included	No corresponding rs number; the variant is excluded as a known single nucleotide polymorphism	Build 155; 2025-12
Human Gene Mutation Database (HGMD)	HTRA1: NM_002775.5; HGVS: p.Asp320Glu	Not included	No disease-related records; not listed in the list of pathogenic/possibly pathogenic variants (DM/DM?)	2024.1; 2025-12
Ensembl Variation	ENSG00000104853 (HTRA1); HGVS: c.960C>G	Not included	No functional annotation or population distribution data; the wild-type base at the corresponding genomic position is C	GRCh38.p14; 2025-12

Retrieval strategy: All databases were searched using HGVS notation (c.960C>G) and corresponding genomic coordinates (GRCh38) of HTRA1 gene (NM_002775.5) to ensure accuracy.

HTRA1 = HtrA serine protease 1.

In this case, the patient’s condition was effectively managed through risk-factor control and lifestyle modification. While this report offers a detailed account of a single patient for reference, the generalizability of the observations remains uncertain. The 1-year follow-up period is relatively short, limiting conclusions regarding long-term prognosis. Studies with larger cohorts and extended follow-up will be required to substantiate and expand upon these findings.

## Author contributions

**Writing – original draft:** Jianzhong Shu, Jinrong Li.

**Writing – review & editing:** Jianzhong Shu.
